# Supplementary material for: Are carbon emissions trading and green financial instruments synergistic? -Comprehensive quantitative research based on content analysis
Source: PLoS One. 2024 Mar 7;19(3):e0298601. doi: 10.1371/journal.pone.0298601 (PMC10919601; doi:10.1371/journal.pone.0298601)
Supplement: S1 Data — (ZIP) [file pone.0298601.s001.zip › Data/地级市Z_F工作报告.zip!/╔┬╬≈/╔╠┬σ╩╨/└┤╫╘╣1⁄2╓┌║┼í╛┬φ┐╦╩2╛▌═°í┐_2010.docx]

各位代表：

　　现在，我代表市人民政府向大会作工作报告，请予审议，并请市政协委员和其他列席人员提出意见。

　　一、2009年工作回顾

　　过去的一年，是新世纪以来我市经济社会发展困难最多、挑战最大的一年。面对严峻复杂的经济形势，我们在省委、省政府和市委的正确领导下，坚持以科学发展观统领经济社会发展全局，认真落实保增长、保民生、保稳定的各项政策措施，科学谋划，化危为机，锐意进取，顽强拼搏，完成了市二届人大四次会议确定的各项任务，继续保持了经济较快向上发展的良好势头。

　　全市实现生产总值225.12亿元，增长14.1%。全社会固定资产投资220.34亿元，增长33.8%。财政总收入16.44亿元，增长15.3%，其中地方财政收入8.96亿元，增长31.3%。城镇新增就业10902人，登记失业率3.6%。城镇居民人均可支配收入12857元，农民人均纯收入3002元，分别增长23.1%和20.5%。社会消费品零售总额72.8亿元，增长19.7%。人口自然增长率控制在2.5‰。

　　项目建设成效显著。争取中省投资项目836个、资金16.7亿元。落实项目前期经费6388万元，完成重大项目前期工作100个。实施的100个市级重点项目完成投资122亿元，比亚迪公司100兆瓦太阳能电池、九龙矿业公司钼采选等重大项目建成投产，金岭金矿东沟选厂生产线投料试车一次性成功，延长氟硅产业园区、西部鑫兴公司钼业深加工等项目顺利推进，商漫高速公路建成通车，西商高速二线、西康及西合铁路增建二线开工建设。新修改造地方公路3176.6公里。330千伏张村变增容改造工程全面启动，440个低压改造工程建成运行。市区5条道路改造、丹江公园二期、江南惠民小区廉租房工程全面完工，龟山风景区、丹江立交桥、市职业技术学院新校区、市体育中心等工程加快实施，中心城市已创建成为省级园林城市。六县一批扩容提质美化工程取得新进展，8个省级重点镇建设规划全面完成。修建基本农田8.62万亩，发展节水灌溉3.66万亩，开发整理土地3.5万亩，治理土地4.1万亩。

　　特色产业快速发展。大力发展“一村一品”，加快推进产业化经营，新建药源基地16万亩、林果基地14.79万亩，发展茶叶3.2万亩、烤烟11.08万亩、设施蔬菜6560亩，新增规模养殖场户2107户；劳务输出50.6万人，创收28.9亿元；粮食总产67.6万吨，增长13.8%。制定并落实了扶持工业经济发展的一系列措施，推进工业企业调结构、扩产能、提效益，现代材料、现代中药、绿色食品三大支柱工业产值占工业总产值的82%，比重较上年提高了13个百分点；规模以上工业完成增加值32.2亿元，增长15.1%。出台了《关于加快旅游产业发展的实施意见》，旅游产业快速发展，消费市场日趋活跃，全市接待游客733万人次，实现综合收入23.1亿元，分别增长61.8%和120.8%；金融机构各项存款余额319.96亿元、各项贷款余额131.9亿元，分别增长24.83%和35.4%。

　　循环经济扎实推进。出台了《关于大力发展循环经济的决定》，编制了《现代材料工业基地建设规划》、《商丹循环工业经济园区总体规划》和《控制性详细规划》，商丹循环工业经济园区被列为省级园区，首期规划的5个循环产业链已有4个启动建设。节能减排强力推进，市污水处理厂一期工程建成投运，医疗固体废物处理厂基本建成，洛南、商南、镇安、柞水、山阳五县污水处理厂启动建设，淘汰落后水泥产能40万吨，推广高效照明产品20万只，建成沼气池1.73万口，万元生产总值能耗降低4.64%，化学需氧量和二氧化硫排放量分别降低4.05%和2.6%，中心城市空气质量达到或好于二级的天数355天。生态建设成效明显，完成造林绿化51.27万亩，治理水土流失1029.38平方公里。

　　改革开放不断深入。行政机构、集体林权、土地流转、财政体制、医药卫生和事业单位等各项改革进展顺利。我市与国家开发银行、中国农业银行等金融机构，与西北农林科技大学、西北有色金属研究院、西北有色地质勘查局等高等院校、科研院所建立了战略合作关系，马来西亚客商在商洛设立了陕西首家外商独资投资性公司。成功举办了第二届中国核桃大会暨首届商洛核桃节，组团参加了西洽会、农高会等大型招商活动，赴北京、天津、上海、深圳等地叩门招商14次，全年招商引资到位资金63.99亿元，实际利用外资4014万美元，分别增长66.55%和52.44%。对外贸易高速增长，外贸进出口总额1.04亿美元，增长289.8%。

　　社会事业全面进步。教育事业均衡发展，教育质量稳步提高，高考二本以上上线5581名，上线率继续位居全省第一；启动实施了“蛋奶工程”，受惠学生15.65万人。科技创新步伐加快，实施科技成果转化和推广项目40个。农村三级医疗卫生服务体系逐步健全，新农合参合率96.9%，继续保持全省第一。公共文化服务体系不断完善，新建农家书屋147个、农村文化活动室170个、乡镇文化站31个。广播电视事业快速发展，建成村级广播电视室534个，新发展光前端有线电视2679户，整体转换数字电视6025户。成功举办了全市第二届运动会。人口和计划生育工作得到加强，为45.17万农村育龄妇女进行了健康检查，全市人口出生率8.75‰，出生人口政策符合率98.71%。

　　 民生民本持续改善。完成民生八大工程投资20.6亿元，其中实施基本建设类项目1454个，投资13亿元。承诺的10件实事全部完成。各类保险覆盖面继续扩大，各项社保基金按时足额支付。城乡低保实现应保尽保，农村五保对象集中供养率达到30%。为残疾人办了10件实事。建设经济适用房20.3万平方米、廉租房25.6万平方米，为3080户低收入住房困难家庭发放租赁补贴549万元。解决农村人口饮水困难和饮水安全18.38万人。启动建设了53个新农村建设示范村、115个扶贫重点村，搬迁贫困人口1741户7431人，筹措产业发展资金1960万元，投放扶贫贴息贷款2.13亿元，扶持贫困户14598户，解决了11.43万贫困人口的脱贫问题。“平安创建”成效明显，安全生产形势基本平稳，人民群众对社会治安的满意率继续位居全省第一，我市被评为全省社会治安综合治理先进市。

　　政府效能全面提升。主动接受人大和政协的监督，先后7次向市人大及其常委会报告工作，6次向市政协通报重点工作进展情况，办理人大代表建议120件、政协委员提案253件，按期办复率达到100%。进一步规范了行政审批程序，废止规范性文件210件、修改64件。积极开展依法行政示范县和示范单位创建活动，“五五”普法、依法治市和政务公开工作深入推进。在全省率先建立了突发事件趋势研判制度，应急管理水平进一步提高。加强重点领域、重大项目的审计监督和行政监察，深入开展治理商业贿赂和小金库专项行动，反腐倡廉工作成效明显。扎实开展“政府工作落实年”活动，制定了市政府目标责任考核暂行办法，干部作风明显改进，行政效能不断提升。

　　统计、税务、地震、气象、民族、宗教、侨务、邮政、通讯、烟草、盐务、老龄、方志、档案、人防、慈善、红十字会、食品药品监管等工作都取得了新的成绩。

　　各位代表，过去的一年，在大事多、难事多的情况下，商洛发生了许多令人振奋的新变化。这些成绩的取得，是市委正确领导的结果，是市人大、市政协监督支持的结果，是全市人民团结奋斗的结果。在此，我谨代表市人民政府，向全体人大代表、政协委员，向奋战在各条战线的工人、农民、知识分子和干部职工，向各民主党派、工商联、人民团体、各界人士和离退休老同志，向驻商部队指战员、武警官兵和公安干警，向支持商洛发展的各界朋友，致以崇高的敬意和衷心的感谢！

　　在看到成绩的同时，我们也清醒地认识到，全市经济社会发展和政府工作还存在一些困难和问题，主要表现在：农产品加工龙头企业少，农业产业化进程缓慢，农民持续增收难度增大；工业结构不尽合理，整体实力不强，抵御风险的能力较差；中小企业发展的瓶颈制约多，非公有制经济发展水平不高，第三产业发展滞后；科技创新体系不健全，自主创新能力不强，科技带动力较弱；骨干财源偏少，财政自给率低，发展资金严重匮乏；城镇化水平较低，尤其是小城镇发展缓慢，承载工业化和拉动内需的能力不足；投资环境有待优化，部分干部群众思想不够解放，少数部门和单位的工作效率不高，等等。对此，我们将以高度负责的精神，采取有力措施，认真加以解决。

　　二、2010年工作目标

　　今年是我市完成“十一五”规划的最后一年，也是全面实施关中-天水经济区发展规划的重要一年。综观经济社会发展态势，尽管金融危机的影响还在持续，但今年我市经济发展环境将好于去年，面临的新机遇和有利因素很多。一是国家继续实施积极的财政政策和适度宽松的货币政策，保持宏观经济政策的连续性和稳定性，这是支撑经济发展的最大利好；二是《关中-天水经济区发展规划》将商洛作为“一核一轴三辐射”中的一个次核心城市，使商洛的发展提升到了国家战略的层面，这是一个千载难逢的重大历史机遇；三是国家将出台西部大开发新的十年规划，省上制定了12个产业调整和振兴规划以及《陕南循环经济产业发展规划》，工作重点由“打基础”转向“兴产业”，为我们加快优势资源深度转化带来了机遇；四是随着境内高速公路建设步伐的加快，我市已成为陕西省高速公路密集区，区位、资源、市场等要素成本优势不断上升，加快发展的环境和条件日益趋好；五是比亚迪、中金、延长、华电、国电、陕西有色等一批国内500强企业相继落户商洛，一批投资过亿元的重大项目相继建成或开工建设，为我市实现率先突破发展奠定了坚实基础。面对新形势、新机遇、新变化，我们必须保持清醒头脑，坚定信心，把握大局，借力而为，乘势而上，努力使全市经济综合实力有一个更大的提高，发展方式有一个更大的转变，区域竞争力有一个更大的提升。

　　今年经济社会发展的主要预期目标是：生产总值增长14%左右；全社会固定资产投资完成280亿元，增长27%；财政总收入完成19.1亿元，增长16%，力争达到20亿元，其中地方财政收入完成10.4亿元，增长16%；社会消费品零售总额完成87亿元，增长19%；城镇居民人均可支配收入14914元，增长16%；农民人均纯收入3602元，增长20%；单位生产总值能耗、化学需氧量和二氧化硫排放量控制在省上下达的指标之内；城镇登记失业率控制在4.5%以内；居民消费价格涨幅3%左右；人口自然增长率控制在5.5‰以内。

　　做好今年的工作，必须把握好以下几点：一是必须坚持项目带动，以项目聚合投资壮大产业，构筑加快发展的坚实基础；二是必须坚持率先发展工业，调整优化工业结构，扩张工业经济总量；三是必须坚持发展循环经济，大力推进技术创新，不断延长产业链条，努力实现产业发展低碳化、绿色化；四是必须坚持城镇带动，扎实推进城乡统筹，加快城乡一体化发展；五是必须坚持改革开放，创新体制机制，优化发展环境，提高招商引资水平；六是必须坚持以人为本，增强公共服务能力，努力保障改善民生，促进社会和谐进步。

　　三、扎实推进全市经济率先突破发展

　　保持经济快速增长仍然是今年的首要任务。必须突出重点，强力推进，扩大经济总量，提高综合实力。重点抓好七个方面工作：

　　 (一)狠抓项目建设不放松，聚集经济发展后劲

　　继续用好、用足、用活国家扩大内需政策，坚持快上项目、多上项目、上大项目，努力扩大投资，拉动经济增长。

　　强力推动项目建设。加快实施100个市级重点项目，年度完成投资150亿元，其中新建项目40个、续建项目60个。加快37个重大产业类项目建设，力促比亚迪1000兆瓦太阳能电池和200万只汽车安全气囊、商州60万吨高性能辅助性胶凝材料、镇安金岭金矿金龙山矿区生产线等项目建成投产，确保比亚迪1.2万吨电解液、商洛氟硅产业园一期氟系列产品开发、西部鑫兴钼业万吨氧化钼等项目基本建成。加大基础设施项目建设力度，配合做好西合、西康铁路增建二线和西商高速二线、商柞高速公路建设，加快产业路建设。加快新一代多普勒天气雷达建设步伐，确保雷达塔楼主体完工、综合数据楼开工建设。积极推进“气化商洛”工程，完成西安至商洛中心城市的天然气长输管网建设任务。

　　加快融资平台建设。坚持全力以赴“争”、创造条件“融”、千方百计“引”，多措并举筹集建设资金，争取中省项目资金6亿元、招商引资到位资金78亿元。抓好西部天成投资公司落地升级工作，确保市创投公司、中小企业担保公司正常运行，上半年完成矿业公司组建工作。积极争取省上中小企业发展基金，尽快成立小额信贷公司。建立重大项目扶持基金，引导和带动社会投资。加强与各类商业银行的合作，力争全年新增贷款30亿元。

　　积极策划储备项目。准确把握中省产业政策和投资导向，依托优势资源，做好项目谋划、筛选、论证和评估工作，催生和孵化一批大项目，尤其是产业类项目，不断优化充实项目储备库。今年市级筹措2500万元，各县区安排资金不少于800万元，用于重大项目的策划、论证和招商，全年策划储备重大项目300个，完成100个重大项目前期工作任务，重点做好西安-(商洛)-武汉客运专线、支线机场等重大项目策划和论证，以及高储能钒电池、石煤综合利用和热电厂等项目前期工作，确保可研以上项目滚动保持在100个以上。

　　(二)坚持率先发展工业，提升新型工业化水平

　　牢固树立率先突破发展必须率先发展工业的理念，按照“大项目支撑、大企业引领、园区化承载、集群化发展”的思路，着力调结构、转方式、扩总量、提效益，确保规模以上工业增加值增长17%。

　　全力扶持骨干企业。认真落实各项优惠政策，重点支持比亚迪、中金、国电、华电、延长、陕西有色、陕煤集团等国内500强企业在商发展。优先为大企业、大集团配置优势资源，支持炼锌厂、九龙公司、五洲公司、秦鼎公司、丹水建材、商南豪迪、秀山水泥、大西沟矿业等重点企业扩大生产规模，延长产业链条，推进资源规模化深度开发。扎实开展“企业服务年”活动，着力营造良好环境、破解融资难题，全力扶持中小企业快速发展。鼓励企业重组和产权多元化，支持市内有条件的企业上市融资。

　　提高企业核心竞争力。以增强自主创新能力为核心，以加快推进技术改造为关键，加大工业结构调整力度，促进现代材料、现代中药、绿色食品三大支柱工业优化升级。抓住今年省财政对企业技术中心建设进行补贴、对重大技术创新课题给予资金支持的政策机遇，依托省上“13115”科技创新工程，启动我市“51158”科技带动工程，重点抓好商洛炼锌厂、香菊公司、盘龙公司等企业的技术中心建设，继续支持企业与科研院所、高等院校开展合作，加强创新能力建设和新产品研发，提高企业发展的整体水平和市场竞争力。加快发展高新技术产业，积极申报国家新材料特色产业基地。

　　推进工业园区建设。加快商丹循环工业经济园区基础设施建设，完成丹南大道、刘湾生态工业园研发区主干道、比亚迪服务区、比亚迪和刘湾大桥工程，不断完善园区配套服务体系，创新园区管理体制机制，提升园区服务、承载和招商能力。加大县域工业园区规划和建设力度，争取丹凤东河生态产业园、山阳县工业园区、商南生态科技产业园等县域园区列入省重点，全年新引进36户企业入园发展。

　　(三)夯实农业农村发展基础，提高“三农”工作水平

　　按照“增收惠民生、强基增后劲”的要求，以建设社会主义新农村为主线，进一步完善农村基础设施，大力发展现代农业，加快农业产业化步伐。

　　大力发展现代农业。认真落实各项强农惠农政策，积极开展粮食高产创建活动，确保粮食总产55万吨左右。抓好108个省级“一村一品”示范村建设，加快实施畜牧业收入倍增、万亩设施蔬菜增收工程，新建畜禽养殖小区15个，发展规模养殖场户3500户，新发展药源基地10万亩、设施蔬菜6000亩。抓住省上支持发展核桃产业基地县的机遇，充分发挥中国•商洛核桃研究所的作用，进一步扩大优质良种核桃发展规模，新建核桃等林果基地35万亩，良种嫁接改造30万亩。加快农产品质量安全监管体系建设，加强特色农产品基地认定和地理标志登记保护，认证绿色、有机和无公害农产品20个，打造商洛特色农产品品牌。坚持就地转移和异地输出并举，加强农民工技能培训，提高劳务输出组织化程度，劳务输出51万人，创收30亿元。

　　积极培育龙头企业。坚持整合与招引相结合，落实企业纳税返还、财政贴息补助等扶持政策，发展认定市级以上农业产业化龙头企业7家，依托企业加速茶叶、核桃、板栗等系列产品开发和品牌整合。加强与陕西农产品加工技术研究院等科研院所的技术合作，开展农产品加工技术项目对接和联合攻关，促进农产品加工技术升级和产品创新。支持发展各类农民专业合作组织，健全农产品营销网络体系，使商洛农特产品走进大城市、进入大型超市。

　　加强农村基础设施建设。新建基本农田9万亩，发展节水灌溉3万亩，完成南秦、龙潭、辛岳、鼓楼河等病险水库除险加固任务。改建干线公路100公里，新建通乡油路197公里，改造县乡公路200公里，完成通达工程450公里。加强农村电网建设，重点实施330千伏张村变增容改造和110千伏城区、灵口输变电及黄龙铺变增容扩建工程，完成商南水沟等4个35千伏输变电工程和81个村的低压改造任务。抓好53个新农村示范村和70个扶贫重点村建设，新建移民示范村26个，实现10万贫困人口稳定脱贫。

　　 (四)发展壮大第三产业，扩大城乡消费需求

　　依托区位和资源优势，以生态旅游为龙头，加快发展第三产业，激发社会消费需求，推动经济快速发展。

　　加快发展旅游产业。着眼大旅游、大市场、大产业发展，加快融入关中旅游圈和全国旅游大循环，打造秦岭生态旅游最佳目的地。加大招商引资力度，加快仙娥湖生态旅游度假区开发步伐。坚持注重特色、错位发展，抓好四个国家级森林公园开发升级，牛背梁、天竺山两个公园“十一”开园。把挖掘仓颉、商鞅、四皓、闯王等历史文化与发展旅游结合起来，确保金丝峡、牛背梁等景点演艺进景区。大力发展乡村旅游，发展农家乐200家。建成市、县区旅游咨询服务中心。加快旅游配套服务设施建设，满足游客“吃、住、行、游、购、娱”需求。筹集6000万元资金，强化旅游宣传促销，积极开发旅游新产品，不断提升商洛旅游知名度，全年接待游客900万人次，实现综合收入30亿元。

　　着力发展现代服务业。大力支持餐饮服务业发展，通过招商启动建设8个四星级以上酒店，提升接待服务水平。按照大物流、大交通的目标，积极引进国内外物流龙头企业，加快商山物流园建设步伐，构建完善的物流网络体系，把我市建成陕西重要的物流集散地。积极培育保险市场，制定出台支持发展会计、法律、咨询、评估、策划、创意等新兴服务业的具体政策和措施，不断提升现代服务业的发展层次。坚持政府引导、市场运作，鼓励有资质、有实力、有信誉的大企业投资房地产开发，建设一批规模较大、档次较高、配套齐全的住宅小区，全市完成房地产投资8亿元。

　　进一步扩大城乡消费。大力开发社区商业、物业、家政等服务性消费，扩大城市居民日常消费。继续做好家电、汽车、摩托车、农机具下乡工作，进一步激活农村消费。积极培育休闲、假日、娱乐、保健等消费热点。按照大流通、网络化、全覆盖的要求，继续实施“万村千乡市场工程”、“新网工程”、“双百市场工程”、“县超工程”和“农超对接工程”，建成7个县级配送中心、13个农产品批发交易市场，建设和改造400个标准化农家店，完成5个五级客运站建设任务。加强市场监管，维护市场秩序，营造便利、安全、放心的市场消费环境。

　　 (五)推进城镇化建设，加快城乡一体化进程

　　加强城镇规划、建设和管理，打造以中心城市为核心、以各县县城为重点、以小城镇为基础的相互衔接、梯次推进、协调发展的格局，进一步提高全市城镇化水平。

　　突出规划引领。以建设关中-天水经济区次核心城市、西安第二生活区、打造山水园林生态旅游城市为目标，按照“北上、南扩、东进、西优”和推进商丹一体化的发展思路，完善中心城市总体规划，完成环城北路控制性详规编制任务。高标准编制六县县城发展规划和村庄建设规划，提高城镇控制性详规覆盖率。加强各类规划衔接和管理，严格按规划建设项目。

　　加快城镇建设。充分发挥市城投公司的作用，多渠道筹集建设资金，抓好环城北路、商鞅大道、龟山风景区二期、工农路改造、西街和莲湖公园东侧旧城改造、莲湖公园南扩、江滨大道北侧开发、丹南新区基础设施、行政中心片区等重点工程建设。加大城市绿化工作力度，实施城周绿化1.5万亩，其中投资1800万元，完成市区和龟山、金凤山等区域绿化5000亩。扎实推进商南、镇安两县创建省级园林县城工作。加快6县县城和8个重点镇建设步伐，提高城镇辐射带动能力。

　　强化城市管理。进一步完善城市管理机制，加快中心城市村改居步伐，提高城市管理水平。不断深化户籍制度改革，加快农村人口向小城镇集中，不断增强城镇的综合承载能力。抓好商州区统筹城乡发展试点工作，加快城乡经济社会一体化发展。充分运用市场化手段经营和管理城市，促进特色产业、优势项目向县城和重点镇集聚，大力发展城镇经济，实现城镇滚动发展。

　　扎实推进创卫。按照年内中心城市“创卫”达标的总体要求，加快完善市政公共服务设施，重点抓好公厕、集贸市场、环卫设施、建筑立面改造等基础设施建设，确保6月底前全面完成建设任务。坚持集中整治和常态管理相结合，扎实开展建筑工地、集贸市场、市容市貌、交通秩序、门头牌匾、背街小巷环境、烟尘和噪声、医疗卫生、“五小”行业等专项整治，坚决依法拆除和取缔占道经营、店外经营和流动摊点。加大创卫宣传力度，充分调动广大群众参与创建的积极性和主动性。深入开展健康意识教育，引导群众养成健康、科学、文明的生活方式和良好的行为习惯。积极推动市县联创，抓好商南、丹凤两县创建省级卫生县城工作。

　　 (六)深入推进各项改革，提高开放开发水平

　　坚定不移地推进各项改革，紧跟大企业、大集团战略投资和产业梯度转移趋势，在大区域、大市场中谋求新的发展。

　　深入推进改革创新。全面完成市县区两级政府机构改革任务，稳步推进乡镇机构改革。继续对财政产业性资金的投入方式进行改革，逐步建立以贴息、担保、参股和风险投资为主的投入机制，充分发挥财政资金的杠杆作用。扎实推进农村土地承包经营权流转，引导家庭经营向生产集约化发展。加快推进集体林权制度改革，真正将林地承包经营权和林木所有权落实到农户。进一步扩大农业保险覆盖面，切实为农业产业化发展提供保障。加快推进供销社改革，强化现代经营服务功能。积极推行基本药物和药品“三统一”制度，抓好省级医疗卫生综合改革试点工作。继续抓好行政事业单位资产管理改革、文化体制改革和科研机构体制改革。

　　努力扩大对外开放。下大气力整治投资环境，努力打造低成本、高回报的投资“洼地”。坚持把招商引资作为对外开放的首要任务，突出大招商、招大商，主动加强与国内500强企业的合作，争取引入中建集团、陕煤集团等大企业、大财团投资商洛。积极承接产业梯次转移，面向南京等友好城市，以及“长三角”、“珠三角”、“环渤海”和港澳台地区，组织招商小分队叩门招商，积极组团参加西洽会等各类节会招商，大力开展以商招商和产业链招商，吸引更多的客商来商洛投资兴业。完善重大签约项目跟踪落实机制，确保合同落实、项目落地。主动加强与关中-天水经济区内各市的区域合作，促进优势互补、共同发展。进一步加强对外交流，发展国外友好城市。大力发展进出口贸易，力促外贸进出口快速增长。

　　大力推进全民创业。认真实施全民创业计划，扩大小额贷款、信用贷款额度，支持中小企业和个体创业者加快发展，支持商洛籍在外企业家回商投资兴业，支持在外务工致富人员返乡创业，支持农村能人创办企业，全年新增私营企业240户、个体经营户2100户。强化和落实促进就业的政策措施，确保新增城镇就业10000人，实现下岗失业人员再就业4600人、就业困难对象再就业800人。健全非公有制经济发展的体制机制，进一步放宽市场准入条件，促使非公有制经济快速发展，全年非公有制经济增加值增长15.5%。

　　 (七)加快发展循环经济，转变经济发展方式

　　认真贯彻《关于大力发展循环经济的决定》，强化节能减排措施，加快形成节约资源和保护环境的产业结构、增长方式和消费模式，增强可持续发展能力。

　　大力发展循环经济。加快落实商丹循环工业经济园区规划项目投资主体，努力把商丹循环工业经济园区打造成为我省循环经济发展的示范区。扎实搞好商洛炼锌厂循环经济发展试点，积极推进县区循环工业经济园区试点，促进重点行业、园区和农村循环经济快速发展。制定矿业深度开发实施意见，加大钼、钒、铁、金、硅等优势矿产资源探矿权、采矿权整合力度，推进矿产资源有序开发、循环利用。加强尾矿综合利用工作，编制完成《尾矿综合利用规划》，重点抓好商洛炼锌厂10万吨电解锌废渣、镇安鑫安建材公司金尾矿蒸养砖等尾矿综合利用项目建设，最大限度地提高资源利用率，力争将我市建成全国尾矿综合利用示范基地。围绕大型环保和节能装备、资源综合利用设备、新型洁净产品等领域，加快制定环保产业发展规划，明确发展重点、需要突破的核心技术以及相应的政策支持措施。充分发挥陕西材料工程研究院商洛分院的作用，加快推进技术与成果转化，为发展循环经济和新材料基地建设提供有效支撑。

　　扎实推进节能减排。抓好15个节能减排项目建设，抓紧实施丹凤葡萄酒公司废水治理项目，加快六县污水处理厂和垃圾处理厂建设进度，丹凤县污水处理厂必须完成50%以上工作量，洛南、商南、镇安、柞水、山阳五县污水处理厂必须完成建设任务。大力推广农村生活垃圾“户分类、村收集、乡运输、县统一处置”模式，切实加强农村环境综合治理。积极推广节能新技术、新工艺，加快建筑节能技术的推广和应用。大力发展农村清洁能源，完成省上下达的农村沼气建设任务。完善市区污水排放管网，提高城市污水收集率和处理率。加大城市供水管网改造力度，推广农业节水技术，节约生产生活用水。继续调整优化用地结构，搞好农村“空心村”治理和土地整理工作，盘活土地存量，节约集约用地。

　　加强生态环境建设。继续抓好天然林保护、退耕还林等重点工程建设，进一步提高森林碳汇，完成工程造林35万亩、重点区域绿化2.5万亩。以国家南水北调中线工程水源涵养区、汉丹江流域商洛段环境综合整治为重点，完成“丹治”60条小流域治理任务。加快中小河流综合治理步伐，新修加固堤防200公里，防治水土流失1000平方公里。

　　四、积极推动和谐商洛建设

　　坚持统筹经济社会发展，更多地增加社会事业投入，更好地关注和改善民生，更有力地推进社会和谐稳定。

　　 (一)协调发展各项事业，推动社会全面进步

　　坚持教育优先发展。大力发展学前、基础、职业、特殊、成人等各类教育事业，促进义务教育均衡发展，完成商州区义务教育均衡发展合格区创建任务，全面启动山阳县“双高普九”工作。加快商洛职业技术学院新校区宿舍楼、教学楼建设，力争秋季开学投入使用。大力实施校舍危房改造工程，维修、加固、重建校舍26万平方米。加强学校文化建设，强化教师队伍培训，深化新课程改革，全面实施素质教育，不断提高教育教学质量。认真落实义务教育经费保障政策，让所有适龄儿童免费入学。

　　重视发展卫生计生事业。加快市中医院医技综合楼建设步伐，完成市中心医院住院大楼主体工程和规范化村卫生室建设任务，逐步改善各级医疗卫生条件。加强公共服务体系建设，抓好全市65岁以上老人健康检查工作，完成城乡居民健康档案建档任务。规范新型农村合作医疗管理，受益面由70%提高到80%。建立健全联防联控机制，强化重大疾病预防控制。继续加强人口和计划生育工作，认真落实各项奖励扶助政策，积极实施优生促进工程、计生家庭创业工程和农村母亲健康工程，全面推行农村育龄妇女免费健康检查治疗，加强流动人口管理与服务，确保稳定低生育水平、提高人口素质、出生人口政策符合率达到95%以上。

　　进一步加强人才工作。制定出台引进人才的具体优惠政策，吸纳一批中高级实用人才。整合各类培训资源，落实人才培训计划，针对比亚迪、延长、中金等大企业、大集团用人需求，加强专业技术、企业管理和实用人才培训，为企业提供良好的人才服务。加强基层教师、医护人员的培养和交流，提高其专业素质与基本技能。建立健全人才激励机制，激发各类人才的创业热情。

　　加快文体广电事业发展。大力实施“三馆一院一站一室一屋”工程，维修改造2个县区文化馆、1个图书馆、1个博物馆、1个剧院，新建20个乡镇文化站、110个村级文化活动室、305个农家书屋。继续抓好文化遗产保护工作。加快市体育中心建设进度，完成市体育馆和体育场主体工程建设任务，启动建设市游泳馆。重视发展广播电视事业，解决840个20户以上自然村通广播电视问题，新发展光前端有线电视5000户，整体转化数字电视4000户，启动建设手机电视移动工程。同时，抓好100个农村社区建设。完善村务公开民主管理和村民议事日制度，保障农村居民的民主权利和合法权益。

　　 (二)大力实施民生工程，着力保障改善民生

　　 增强公共财政保障能力。落实中省市支持县域经济发展的财税政策，加大骨干财源建设力度，提高财政自给率。加强税收征管，确保应收尽收。完善国库集中支付、行政事业单位资产管理、资金整合等财政管理体制机制，在市本级逐步推行公务卡制度。强化预算执行，压缩会议费、招待费等开支，重点保障义务教育、各类补助补贴等民生方面投入。加强对民生资金和民生工程的审计监督，确保项目资金安全、有效使用。

　　深入实施民生八大工程。加大民生项目争取力度，足额落实市县配套资金，全年完成民生八大工程投资24.5亿元，其中实施基本建设类民生项目1420个，完成投资15.5亿元，增长19.2%。全力为民办好十件实事：一是全面实施“蛋奶工程”。为18.4万义务教育阶段寄宿制中小学和农村幼儿园在校生提供营养早餐。二是启动建设市特殊教育学校。在市区启动建设一所兼文化与职业教育为一体的特殊教育学校，使全市听障、视障和智障儿童能够接受良好的国民教育。三是推行城镇职工医疗市级统筹。将城镇职工、居民住院医疗费用报销比例分别提高到70%和50%以上。四是提高城乡低保最低保障标准。城市低保最低生活保障标准由每月人均160元提高到180元，农村低保最低保障标准由每月人均50元提高到60元。五是加强农村五保供养工作。新建4个县区中心敬老院、8个乡镇敬老院，改扩建3个乡镇敬老院，五保对象集中供养率由30%提高到40%以上。六是加快保障性住房建设。新建廉租房30万平方米、经济适用住房20万平方米，改造农村危房2万户，逐步解决乡镇机关和教育、卫生等事业单位干部职工以及城镇中低收入家庭的住房困难。七是扩大企业养老保险覆盖面。完成企业养老保险扩面3800人，将养老金标准由1060元提高到1120元。八是扩大农村养老保险试点。确保15万农民享受农村养老保险政策。九是加快实施农村饮水安全工程。投资1亿元，建设农村饮水工程236处，解决农村饮水困难和饮水安全16万人。十是强化农村信息服务。建成607个农村信息综合服务站。同时，高度重视做好老龄、青少年和妇女儿童权益保障工作，加快发展社会福利和慈善事业，扎实做好特困残疾人定期定额生活补助工作，让广大人民群众共享改革发展成果。

　　 (三)加强社会管理，确保全市社会大局稳定

　　认真抓好信访工作。严格落实各级领导干部信访工作责任制和责任追究制，切实解决土地征用、搬迁安置、涉法涉诉等涉及群众切身利益的突出问题。继续开展领导干部定期接访、下访、约访、回访和信访“三无”县区、“三无”乡镇创建活动。实行严格的信访工作考核机制，确保实现“一控三降”目标。

　　强化安全生产监管。认真落实各项安全生产制度，强化企业安全生产主体责任和行业安全监管责任，重点抓好民爆烟爆、非煤矿山、尾矿库、工程施工、道路交通、食品药品、公共聚集场所等重点行业和领域的隐患排查，有效防范和遏制重特大事故发生。

　　全力维护社会稳定。完善应急预案体系，加强应急队伍建设，强化基层应急管理，提高防控突发事件能力。加大社会治安综合防控力度，依法严厉打击黑恶势力、严重暴力、多发性侵财和涉毒等违法犯罪活动。深入开展“平安商洛”创建活动，推进社会管理创新，扩大“平安社区”、“平安村镇”、“平安企业”创建覆盖面，进一步提高人民群众的满意度。

　　在抓好经济社会发展工作的同时，围绕全市经济社会发展基础性、全局性和战略性重大问题，结合《关中-天水经济区商洛区域发展规划》，广泛组织开展调研和论证，认真编制全市“十二五”规划，明晰今后五年的基本思路和发展目标，策划大项目、培育大产业，搞好大配套、形成大格局，将商洛的发展引入科学发展、率先突破发展的快车道。

　　五、努力建设人民满意政府

　　新的形势、新的任务，对政府工作提出了更高的要求。我们将进一步加强自身建设，提高行政效能，为人民服务，对人民负责，受人民监督，让人民满意。

　　不断解放思想，增强施政力。深化大学习、大调研活动，引导各级干部注重学习借鉴，注重调查研究，注重工作实践，增长新知识，树立新观念，适应新形势，增强敏锐洞察、见微知著的判断能力，增强统筹兼顾、果断理性的决策能力，增强锐意创新、争创一流的创造能力，增强应对危机、因势利导的掌控能力，不断提高工作的预见性、措施的有效性、方法的灵活性。

　　规范行政行为，增强公信力。严格按照法定权限和程序履行职责，加快推进政府工作法制化。完善重大事项集体决策、专家咨询、社会公示和听证制度，推进科学、民主和依法决策。自觉接受人大依法监督和政协民主监督，切实加强群众监督、舆论监督和政府内部监督。认真办理人大代表议案、建议和政协委员提案，密切与各民主党派、总工会、工商联、共青团、妇联会等人民团体的联系沟通。加强政府信用建设，努力提高政府的公信度。

　　完善落实机制，增强执行力。加快推进政府效能建设，进一步完善政府工作落实机制，不断强化任务分解、定期汇报、督办检查、公开通报、考核奖惩等五项制度，确保既定的工作不折不扣、落到实处。切实精文简会、减少应酬，简化办事程序，提高工作效率。坚持问政于民、问需于民、问计于民，利为民谋，事为民做，卓有成效地完成各项目标任务。

　　加强廉政建设，增强凝聚力。认真贯彻《中共中央关于进一步从严管理干部的意见》，建立健全严格教育、强化预防、及时发现、严肃纠正的工作机制和制度，建设人民满意的公务员队伍。继续强化对权力的制约和监督，进一步深化领导干部任期内经济责任审计。严格执行《中国共产党党员领导干部廉洁从政若干准则》，认真落实党风廉政建设责任制，狠刹部门和行业不正之风，坚决惩处腐败行为。厉行勤俭节约，降低行政成本，树立廉政勤政的政府形象。

　　各位代表，新的一年，任务艰巨，责任重大，使命光荣。让我们高举中国特色社会主义伟大旗帜，以邓小平理论和“三个代表”重要思想为指导，深入贯彻落实科学发展观，在市委的坚强领导下，矢志艰苦奋斗，勇于开拓创新，在推进商洛率先突破发展、全面建设富裕文明和谐新商洛的伟大征程中，迈出新步伐，谱写新篇章！
